# Towards Precision Medicine in Spinal Surgery: Leveraging AI Technologies

**DOI:** 10.1007/s10439-023-03315-w

**Published:** 2023-07-14

**Authors:** Aaron Lawson McLean

**Affiliations:** Department of Neurosurgery, Jena University Hospital – Friedrich Schiller University Jena, Am Klinikum 1, 07747 Jena, Germany

**Keywords:** Spinal surgery, Artificial intelligence, Surgical decision making, Healthcare technology, Data security, Patient outcomes

## Abstract

This critique explores the implications of integrating artificial intelligence (AI) technology, specifically OpenAI’s advanced language model GPT-4 and its interface, ChatGPT, into the field of spinal surgery. It examines the potential effects of algorithmic bias, unique challenges in surgical domains, access and equity issues, cost implications, global disparities in technology adoption, and the concept of technological determinism. It posits that biases present in AI training data may impact the quality and equity of healthcare outcomes. Challenges related to the unique nature of surgical procedures, including real-time decision-making, are also addressed. Concerns over access, equity, and cost implications underscore the potential for exacerbated healthcare disparities. Global disparities in technology adoption highlight the importance of global collaboration, technology transfer, and capacity building. Finally, the critique challenges the notion of technological determinism, emphasizing the continued importance of human judgement and patient-care provider relationship in healthcare. The critique calls for a comprehensive evaluation of AI technology integration in healthcare to ensure equitable and quality care.

## Dear Editor,

I am writing to provide a critique and analysis of the recent paper titled “Will ChatGPT/GPT-4 be a Lighthouse to Guide Spinal Surgeons?” by He et al. [1]. While the authors discuss potential applications of OpenAI’s advanced large language model GPT-4 and its chatbot-style interface ChatGPT in spinal surgery, it is crucial to examine the broader implications of technological determinism, biases, challenges in surgical domains, access and equity issues, cost implications, and global disparities in technology adoption within the context of clinical medicine and the biomedical enterprise.

Firstly, it is essential to address the issue of algorithmic bias in AI applications. Algorithms, including language models like GPT-4, can inherit and perpetuate biases present in the data they are trained on. These biases may disproportionately impact marginalized populations, leading to unequal healthcare outcomes [2, 3]. It is crucial to thoroughly evaluate and mitigate biases to ensure fair and equitable treatment for all patients. For example, Ahsen et al.'s study on breast cancer diagnosis demonstrated that biases inherited by algorithms from data generated by humans can diminish performance [4]. However, a bias-aware algorithm integrated into a clinical decision support system was able to mitigate the adverse impact of bias, improving the accuracy of decisions based on mammography. Specific examples of algorithmic biases relevant to spinal surgery must be explored to assess their potential impact on diagnostic accuracy, treatment recommendations, and surgical decision-making. Once identified, mitigating algorithmic biases in AI applications, including language models like GPT-4, is crucial in spinal surgery to ensure equitable care.

Furthermore, when considering the integration of AI technologies in surgical domains, unique challenges arise. Surgical procedures require real-time decision-making, precise manual dexterity, and adaptability to unforeseen circumstances. While ChatGPT/GPT-4 may assist with information retrieval and decision support, its ability to account for dynamic surgical situations and respond appropriately remains unverified. The limitations and challenges faced by AI in surgical domains should be acknowledged, including the need for rigorous validation, addressing technical limitations, and ensuring seamless integration with existing surgical workflows (Table 1).

**Table 1:** The table presents specific examples of potential challenges in integrating ChatGPT/GPT-4 into the practice of spinal surgery, mapped to five main considerations: Algorithmic Bias, Surgical Domain Challenges, Access and Equity, Global Disparities, and Technological Determinism. It illustrates the potential issues, their implications, and how these issues may manifest in the context of spinal surgery.

Consideration	Potential challenge	Example in spinal surgery
Algorithmic Bias	Algorithms can inherit and perpetuate biases present in the data they are trained on.	An AI system might be biased towards recommending surgical interventions over non-surgical treatments if the training data is dominated by records of patients who underwent surgery.
Surgical Domain Challenges	AI technologies may struggle to adapt to dynamic surgical situations.	ChatGPT/GPT-4 might fail to account for intraoperative complications, such as an unexpected bleed or an adverse reaction to anaesthesia, which require immediate and nuanced decision-making.
Access and Equity	Adoption of AI technologies may exacerbate disparities due to the cost of implementation and necessary infrastructure.	In resource-poor settings, the implementation of a complex AI system like GPT-4 might be unfeasible due to lack of technical infrastructure, leading to a widened gap in care quality. Alternatively, smaller clinics may not be able to afford the cost of implementation and training, restricting the technology to more affluent or larger healthcare systems.
Global Disparities	Technological advancements are often concentrated in high-income regions.	Lower-income countries might lack the financial resources and technical expertise to implement and maintain AI systems like GPT-4, leading to disparities in global access to advanced care options.
Technological Determinism	Overemphasis on technology may undermine the essential role of healthcare professionals.	Over-reliance on ChatGPT/GPT-4 might lead to the marginalization of human judgment in spinal surgery, leading to scenarios where essential aspects of patient care and experience are overlooked. This might include considering a patient's personal comfort with a treatment plan, their psychological wellbeing, or their personal values and goals of care.

The discussion of ChatGPT/GPT-4 in spinal surgery cannot overlook access and equity issues. Implementing AI-driven technologies requires robust infrastructure, adequate training, and financial resources [5]. However, access to such resources may be limited in certain regions or healthcare settings, exacerbating healthcare disparities. The potential cost implications associated with the adoption and maintenance of AI systems, as well as training healthcare professionals, should be carefully considered to ensure equitable access for all patients, regardless of socioeconomic status or geographical location.

Global access to advanced technologies like ChatGPT/GPT-4 faces significant obstacles. Technological advancements are often concentrated in high-income regions and prestigious institutions, creating a digital divide that hampers equal access to cutting-edge tools. Developing countries and underserved communities may struggle to overcome barriers related to infrastructure, funding, and expertise. Efforts should be directed toward promoting global collaboration, technology transfer, and capacity building to address these disparities and foster equitable healthcare delivery.

The notion of technological determinism in clinical medicine and the biomedical enterprise must also be critically examined. While AI technologies like ChatGPT/GPT-4 hold promise, they should not be viewed as a panacea for all healthcare challenges. Human judgment, experience, and the doctor-patient relationship remain integral to medical practice. Emphasizing technological determinism can undermine the essential role of healthcare professionals, potentially leading to overreliance on AI systems and neglecting critical aspects of patient care that require human expertise and compassion [6].

In conclusion, I urge a comprehensive evaluation of the potential implications of ChatGPT/GPT-4 in spinal surgery, considering algorithmic biases, challenges in surgical domains, access and equity issues, cost implications, global disparities, and the impact of technological determinism. Responsible integration of AI technologies in healthcare should prioritize patient well-being, address biases, ensure equitable access, and maintain the essential role of healthcare professionals in decision-making and patient care.

Sincerely,

Aaron Lawson McLean
